# Taurochenodeoxycholic Acid Inhibited AP-1 Activation via Stimulating Glucocorticoid Receptor

**DOI:** 10.3390/molecules24244513

**Published:** 2019-12-10

**Authors:** Lei Li, Chang Liu, Wei Mao, Bayaer Tumen, Peifeng Li

**Affiliations:** 1 Key Laboratory of Quality & Safety Control for Pork, Ministry of Agriculture and Rural, College of Animal Science, Anhui Science and Technology University, Fengyang 233100, China; lil@ahstu.edu.cn; 2 Key Laboratory of Clinical Diagnosis and Treatment Techniques for Animal Disease, Ministry of Agriculture and Rural, College of Veterinary Medicine, Inner Mongolia Agricultural University, Hohhot 010018, China; maowei820115@163.com; 3 Shanxi Animal Disease Control Center, Taiyuan 030027, China; tumenbayaer@163.com

**Keywords:** taurochenodeoxycholic acid, activator protein-1, glucocorticoid receptor, anti-inflammation

## Abstract

Taurochenodeoxycholic acid (TCDCA) as a primary bioactive substance of animal bile has been shown to exert good anti-inflammatory and immunomodulatory functions in adjuvant arthritis in rats. The anti-inflammatory and immunomodulatory properties of TCDCA have exhibited interesting similarities with the effects of glucocorticoids (GCs). To investigate the potential mechanisms of TCDCA in anti-inflammation and immunomodulation, we used a luciferase reporter assay to evaluate the activation of the glucocorticoid receptor (GR) stimulated by TCDCA. Our results showed that GR was activated by TCDCA in a concentration-dependent manner. Moreover, the elevated expressions of c-Fos and phosphorylated c-Jun induced by interleukin-1β (IL-1β) were reversed by TCDCA. The inhibition of TCDCA on the transactivation of activator protein-1 (AP-1) was observed as well. However, the suppression of TCDCA on the phosphorylation of c-Jun was blocked incompletely by GR inhibitor RU486. These results have indicated that the anti-inflammatory and immunomodulatory functions of TCDCA involve multiple pathways, with contributions from GR and its related AP-1 signaling pathway.

## 1. Introduction

**Figure 1:**
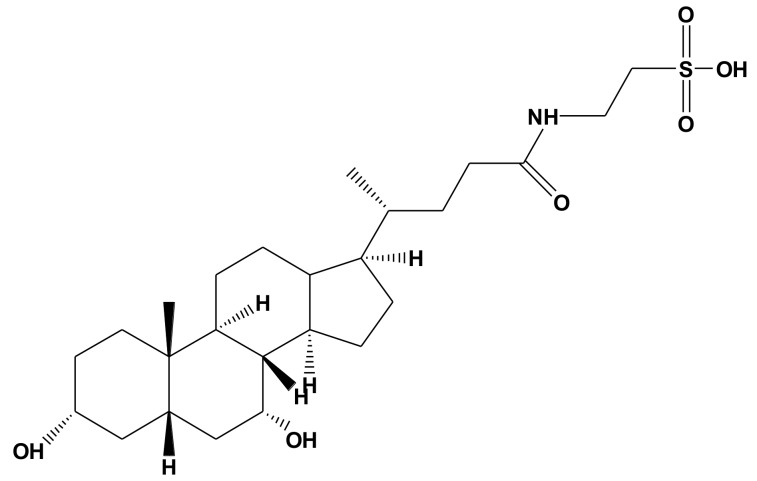
The chemical structure of taurochenodeoxycholic acid.

Taurochenodeoxycholic acid (TCDCA, Figure 1), as a kind of primary bioactive substance of animal bile, is synthesized with taurine (Tau) and chenodeoxychlolic acid (CDCA) in the liver [1]. It is well documented that bile acids (BAs) are crucial in facilitating digestion, absorption, and excretion of dietary lipids, while an increasing number of biological functions of BAs have been discovered in the past few decades. It was found that BAs, as a kind of signal molecule, were involved in numerous cellular signaling pathways, including activating apoptotic, inflammatory and carcinogenic signaling pathways [2,3,4,5,6]. BAs have also been demonstrated to have anti-inflammatory and immunomodulatory effects [4,7,8,9,10,11,12], which mainly operate by activating intracellular ligand-activated nuclear receptors (NRs), such as the farnesoid X receptor (FXR, NR1H4), glucocorticoid receptor (GR) [13,14,15,16], and membrane-type receptors, specifically the G protein-coupled BA receptor (TGR5 or GPBAR-1) [17]. In our previous study, we found that TCDCA could be used to combat adjuvant arthritis by inhibiting the expression of pro-inflammatory cytokines, such as interleukin-1β (IL-1β), IL-6, tumor necrosis factor-α (TNF-α), and induce apoptosis of fibroblast-like synoviocytes (FLS) [7,8,18], which suggested that TCDCA may be a potential therapeutic agent for rheumatoid arthritis (RA) or other inflammatory diseases.

Activator protein-1 (AP-1) as a transcription factor is composed of homo- and/or heterodimers of Jun and Fos proteins [19], with c-Jun-c-Fos heterodimer being the principal form of AP-1. A previous study found that AP-1 played an essential role in the pathogenesis of RA, including synovial hyperplasia and abnormal immune responses [20]. AP-1-regulated IL-1β is the most important cytokine responsible for cartilage breakdown and osteoclastogenesis in RA [21]. IL-1β and AP-1 influence each other’s expression, activity and cross-talk involved in RA joint destruction [21]. Thus, suppression of AP-1 may result in decreasing synovial overgrowth and joint destruction [22]. Therefore, we thought suppression of AP-1 might play a critical role in combatting adjuvant arthritis using TCDCA. In the current study, we observed the influence of TCDCA on AP-1 activation and its mechanisms involved in the anti-inflammatory effects of TCDCA.

## 2. Results

### 2.1. Cell Viability

**Figure 2:**
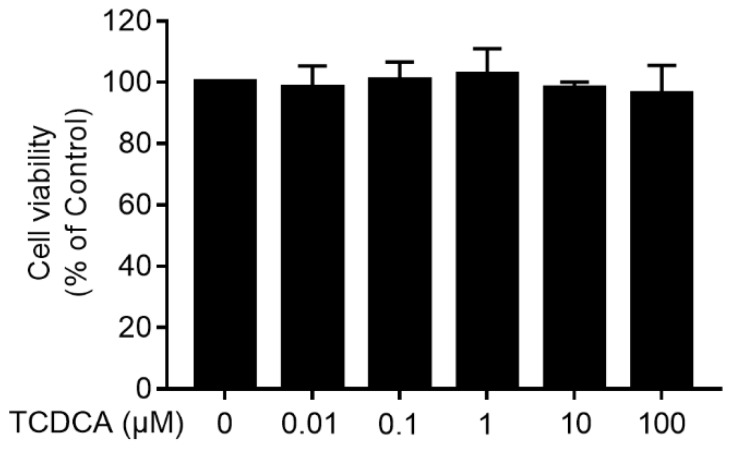
The cytotoxicity of TCDCA on fibroblast-like synoviocytes (FLS). Untreated FLS were used as a negative control. Results were representative of three independent experiments.

To determine the cytotoxicity of TCDCA, an MTT assay was performed. FLS was treated with TCDCA (0.01 μM–100 μM), and cytotoxicity was not observed (Figure 2).

### 2.2. Taurochenodeoxycholic Acid Induced the Transcriptional Activation of Glucocorticoid Receptor

**Figure 3:**
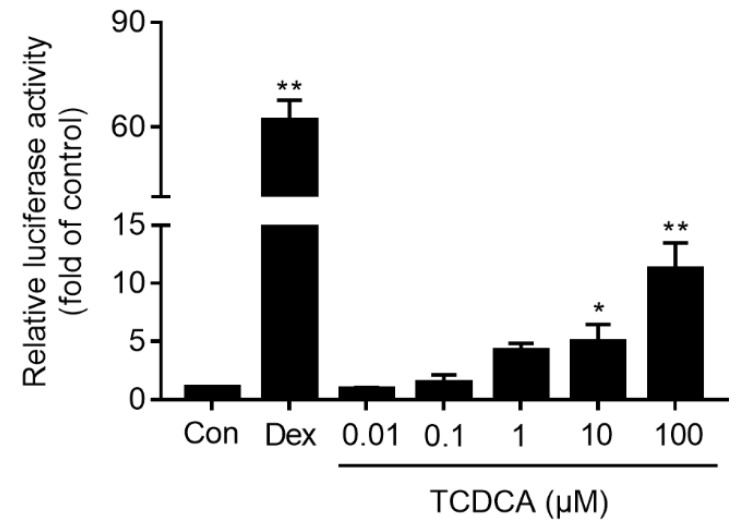
Activation of GR stimulated by TCDCA. Untreated HEK 293t cells were used as the negative control, and Dex (500 nM) was used as the positive control. Results were representative of three independent experiments. * *P <* 0.05, ** *P <* 0.01 vs. control.

To determine the role of TCDCA on the transcriptional activation of GR, a luciferase report assay was used. The results showed that GR was activated significantly by incubating with TCDCA (10 and 100 μM) for 24 h in a concentration-dependent manner compared to the control (*P <* 0.05 or *P <* 0.01). As shown in Figure 3, the maximum fold on the activation of GR by TCDCA (100 μM) was 10.88-fold, and the positive control dexamethasone (Dex, 500 nM) activated GR by a fold of 61.37.

### 2.3. Effects of TCDCA on c-Jun, c-Fos Expression and c-Jun (Ser63) Phosphorylation

**Figure 4:**
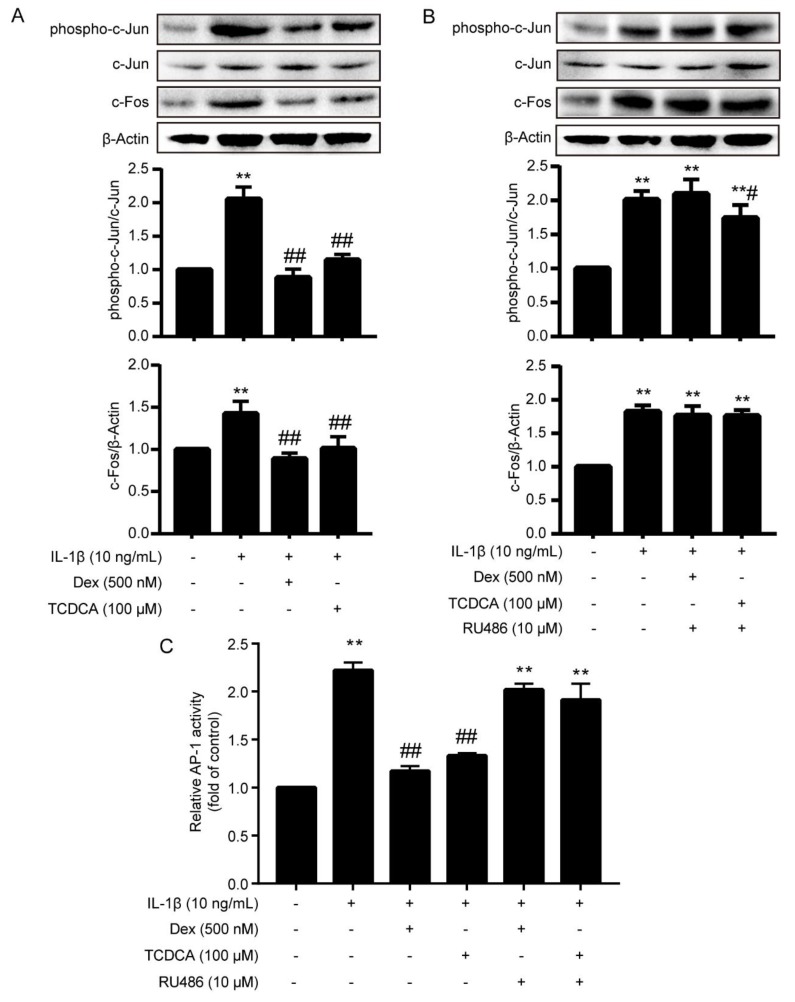
TCDCA inhibited the activation of AP-1. (**A**) Inhibition of c-Jun, phosphorylated c-Jun (Ser63) and c-Fos are detected by immunoblotting using specific antibodies, β-actin was used as a loading control. Untreated FLS was used as a negative control, and Dex was used as a positive control. (**B**) RU486 blocked the suppression of phosphorylation of c-Jun (Ser63) and expression of c-Fos induced by TCDCA. (**C**) TCDCA inhibited AP-1 activity. Results were representative of three independent experiments. ** *P <* 0.01 vs. control, ^#^ *P <* 0.05, ^##^ *P <* 0.01 vs. IL-1β.

Western blot was used to determine the effects of TCDCA on the phosphorylation of c-Jun (Ser63) and expression of c-Fos. As shown in Figure 4A, the phosphorylation of c-Jun (Ser63) and the expression of c-Fos were increased remarkably by IL-1β (10 ng/mL) stimulation compared to the control (*P <* 0.01). TCDCA (100 μM) inhibited the increased phosphorylation of c-Jun (Ser63) and expression of c-Fos induced by IL-1β (*P <* 0.01). Meanwhile, Dex (500 nM), the positive control, also reduced c-Jun (Ser63) phosphorylation and c-Fos expression stimulated by IL-1β (*P <* 0.01), and such repression was much stronger than TCDCA. Furthermore, the diminished expression of c-Fos and phosphorylation of c-Jun (Ser63) by Dex (500 nM) were absolutely reversed by GR inhibitor RU486 (10 μM, Figure 4B). However, RU486 only partially reversed inhibition of phosphorylated c-Jun induced by TCDCA (100 μM). These results indicated that TCDCA could suppress the expression of c-Fos and the phosphorylation of c-Jun (Ser63) and the repression was, at least in part, related to TCDCA-induced activation of GR.

### 2.4. TCDCA Inhibited the Transactivation of AP-1

DNA-binding activity of the AP-1 c-Jun subunit was evaluated by a sensitive multi-well colorimetric assay. After IL-1β stimulation, the DNA-binding capacity of c-Jun in FLS was enhanced noticeably compared to the control (*P <* 0.01, Figure 4C). Dex (500 nM) and TCDCA (100 μM) repressed the enhancement elicited by IL-1β (*P <* 0.01), and the repression was blocked by RU486 (Figure 4C). These observations suggested that TCDCA inhibited the transactivation of AP-1 by activating GR, and indicated that the AP-1 pathway played an essential role in the anti-inflammatory effects of TCDCA.

## 3. Discussion

Glucocorticoids (GCs), as an agonist of the GR, is currently the principle therapeutic agent for RA treatment. The classical GR mediated signaling pathway was the primary mechanism of GCs anti-inflammatory and immunomodulatory actions. The inactive GR resides in the cytoplasm, complexed with the chaperones molecular hsp90 and several immunophilins [23,24,25]. Binding to ligand induces a conformational change in GR and releases GR from the complexed chaperone proteins leading to the exposure of nuclear localization signals and facilitating nuclear translocation. After nuclear translocation, GR may dimerize and the homodimeric GR complex can stimulate or suppress transcriptional responses by binding to glucocorticoid response elements (GRE) or negative glucocorticoid response elements (nGRE). Meanwhile, another pathway is involved in anti-inflammatory effects of GR. Ligand-activated GR can bind to pro-inflammatory transcriptional factors including AP-1 and NF-κB and the protein-protein interactions can repress AP-1 and NF-κB regulated gene transcription [26]. The cross-talk between AP-1 or NF-κB and GR is an essential mechanism for anti-inflammatory and immunomodulatory drugs. Thus, GR is considered to be a critical pharmacological target for anti-inflammatory and immunomodulatory medicine.

Animal bile, as a traditional Chinese medicine, has been widely used for the treatment of inflammatory disease (such as acute tracheitis, winter cough, pneumonia and whooping cough) because of its advantageous anti-inflammatory and immunomodulatory functions. In light of animal bile’s pharmacological effects, it was found that the major bioactive substances of animal bile were BAs, including CDCA, ursodeoxycholic acid (UDCA), TCDCA etc. Our previous study demonstrated that TCDCA showed remarkable inhibition of both acute and chronic inflammation. It especially favorably ameliorated the progressive development and bone destruction of adjuvant arthritis in rats [7]. Moreover, it was found that the anti-inflammatory action of TCDCA was mediated by inhibiting the expression of pro-inflammatory cytokines TNF-α, IL-1β and IL-6, and suppressing the activity of NF-κB [18], which was also inhibited when GR was activated [27]. Considering the interesting similarity between the anti-inflammatory and immunomodulatory effects and the chemical structures between TCDCA and GCs, we predicted that TCDCA may exert its anti-inflammatory effect by stimulating GR related signal pathways. In the present study, the luciferase reporter assay was used to evaluate TCDCA-induced GR transcriptional activation, and we found GR were activated by incubation with TCDCA (10, 100 μM) for 24 h (Figure 2). IL-1β is the predominant pro-inflammatory cytokine associated with rheumatoid arthritis (RA) [28], which has been widely used as a stimulator in RA related studies in vitro [29,30,31,32]. The strategy uses IL-1β to stimulate FLS in anti-arthritis drug discovery, which was used as well in this study. The results showed TCDCA could inhibit the increased expression of c-Fos and phosphorylation of c-Jun in FLS induced by IL-1β (Figure 4A). Furthermore, the transcriptional activity of AP-1 induced by IL-1β was suppressed when FLS was incubated with TCDCA (Figure 4C). These results suggested that the mechanism of TCDCA on anti-inflammation was possibly mediated via inhibiting transcriptional activity of AP-1, which was similar to UDCA [16,33,34,35].

However, the suppression of TCDCA on the phosphorylation of c-Jun (Figure 4B) can be blocked incompletely by RU486. Moreover, the capability of TCDCA in stimulating GR was much weaker compared to Dex (Figure 2). Surprisingly, for all other experiments the difference between Dex and TCDCA was not reflected. These data indicated that there were some other potential mechanisms involved in the anti-inflammatory and immunomodulatory properties of TCDCA. FXR and TGR5, as two principal receptors of bile acids, played a pivotal role in anti-inflammation [36]. Previous studies demonstrated that activated TGR5 could inhibit LPS-induced NF-κB activation but not AP-1 activation [37]. However, activation of FXR resulted in elevating the suppressor of cytokine signaling 3 (SOCS3)-an inducible protein, which suppresses the transcriptional activity of AP-1 [38]. Therefore, the inhibition of TCDCA on AP-1 transcriptional activity may be mediated via TCDCA-activated FXR as well, which requires further investigation.

In summary, we found that TCDCA exhibited its anti-inflammatory and immunomodulatory properties by inhibiting transcription and expression of AP-1 with GR partially contributing to this process.

## 4. Materials and Methods

### 4.1. Reagents

TCDCA was dissociated and depurated from chicken bile as described in our previous study [7,8], with the purity being> 99.5%. RU486 and recombinant IL-1β were purchased from Sigma Chemical Co. (St. Louis, MO, USA). Freund′s complete adjuvant (FCA) was purchased from the Shanghai Institute of Biological Products (Shanghai, China). The Nuclear Extract Kit and TransAM^®^ c-Jun activation kit were purchased from Active Motif (Carlsbad, CA, USA). Phenol red-free Dulbecco’s modified Eagle’s medium (DMEM) was purchased from Life Technologies (Waltham, MA, USA). pGL4.35 [luc2P/9 × GAL4UAS/Hygro], pBIND-GR and Bright-Glo™ the luciferase assay system were purchased from Promega (Madison, WI, USA).

### 4.2. Animals and Induction of Adjuvant Arthritis in Rats

The study was approved by the Animal Ethical Committee of Anhui Science and Technology University (No.007, Approval date: 25 February 2019) and conformed to national guidelines on the care and use of laboratory animals. Adjuvant arthritis models were preformed according to our previously reported method [7,8]. Briefly, thirty male wistar rats (11–13 weeks, 170 ± 10 g) were obtained from the experimental animal center of the Academy of Military Medical Sciences in China. All animals were maintained at a controlled temperature (22 ± 2 °C), and a regular light/dark cycle (7:00–19:00, light), and all animals had free access to food and water. Adjuvant arthritis rats were induced as previously described [39,40]. Briefly, rats were immunized on day 0 by intradermal injection of FCA into the foot pad, containing 10 mg heat-inactive Bacillus Calmette-Guerin in 1 mL paraffin oil, into the left hind paw with 0.1 mL for each rat.

### 4.3. Isolation and Culture of Adjuvant Arthritis FLS

Adjuvant arthritis FLS were isolated as previously described with modification [40]. Briefly, fresh synovial tissues were obtained from three adjuvant arthritis rats under sterile conditions each time. All the synovium tissues were minced with fine scissors, incubated in a plastic flask (Corning, New York, NY, USA) and maintained in Dulbecco’s modified Eagle′s medium (DMEM, Gibco, Waltham, MA, USA) supplemented with 10 mM 4-(2-Hydroxyethyl)piperazine-1-ethanesulfonic acid (HEPES, pH 7.2, Promega, Madison, WI, USA), 15% fetal calf serum (FCS, TBD, Tianjin, China), 100 U/mL penicillin and streptomycin (Gibco, Gaithersburg, MD, USA) 50 mM mercaptoethanol in a humidified 5% CO_2_-containing atmosphere at 37 °C for 7 days. After removal of the synovial pieces, the adherent cells were cultured in the same medium. At 70–80% confluence nonadherent cells were removed and adherent cells were trypsinized, split at a 1:3 ratio and recultured in the same medium. The synoviocytes were used in experiments from passages 3. After three passages, most of the cultured synoviocytes comprised a homogeneous population of FLS.

### 4.4. MTT Assay

FLS were seeded into 96-well plates at a cell density of 5 × 10 ^3^ in 200 μL/well and incubated for 24 h till 80% confluence. Then, cells were incubated with or without TCDCA for another 24 h. At the end of the incubation period, 20 μL 5 mg/mL MTT was added in the medium for a further 4 h incubation, then the medium was removed and 150 μL DMSO was added to dissolve the formazan in the surviving cells. The 96-well plates were gently swirled for 5 min at room temperature. The absorbance was measured at 570 nm with 630 nm as a reference. Cell viability was calculated as the percentage of the control.

### 4.5. Luciferase Assay

HEK 293t cells were seeded at 1 × 10^4^ cells/well in solid white 96-well plates (Nunc, Waltham, MA, USA) in phenol red-free DMEM with 5% bovine calf serum (80 μL/well), under a 5% CO_2_ atmosphere at 37 °C. 50 ng each of pGL4.35[luc2P/9×GAL4UAS/Hygro] vector and pBIND-GR vector in 10 μL lipofectamine^®^ 2000-mediated transfection master mix was transiently introduced into HEK 293t cells. The plates were covered and placed in a tissue culture incubator at 37 °C overnight. 10 μL of 10× induction solutions was added to wells to be induced or control solution to non-induced wells and incubated for 24 h. Luciferase activity was analyzed by using the Bright-Glo™ luciferase assay system. Fold induction was calculated with the luminescence information as follows:

Fold induction = Average relative light units of induced cells/Average relative light units of control cells

### 4.6. Protein Extraction

FLS were seeded into flasks. After 4 h-attachment, cells were incubated with or without TCDCA alone or TCDCA and RU486 (10 μM) for 44 h and then stimulated by IL-1β (10 ng/mL) for another 4 h. After treatments, cells were collected and isolated by using the Nuclear Extract Kit according to the manufacturer′s instructions. The protein concentration was detected with a BCA kit.

### 4.7. Western Blotting

Protein extracts were separated by 12% denaturing polyacrylamide gel electrophoresis and transferred onto polyvinylidene fluoride membranes. After blocking in blocking buffer (Beyotime Biotechnology, Shanghai, China), the membranes were exposed to specific primary antibodies at a concentration of 1:1000 for anti-phospho-c-Jun(Ser63), -c-Jun and -c-Fos (Cell Signaling Technology, Danvers, MA) and 1:3000 for anti-β-actin (Proteintech group, Wuhan, China). Membranes were then washed and exposed to HRP-conjugated secondary antibodies (1:1 000 dilution, KPL, Milford, MA, USA) for 1–2 h, at room temperature, followed by incubation with BeyoECL Plus (Beyotime Biotechnology, Shanghai, China).

### 4.8. Transactivation of AP-1 Assay

After treatment with TCDCA for 48 h, FLS were collected by scraping and centrifugation. Nuclear protein extracts were obtained from FLS by using the Nuclear Extract Kit according to the manufacturer′s instructions. Nuclear protein was stored at −80 °C. The activation of AP-1 was measured using the TransAM^®^ c-Jun activation ELISA kit according to the manufacturer′s instruction. Briefly, 5 μg of nuclear protein samples were incubated for 1 h in a 96-well plate immobilized with an oligonucleotide containing a TRE (5′-TGA GTC A-3′), to which phosphorylated c-Jun contained in nuclear extracts specifically binded. After washing, phospho-c-Jun antibody (1:500 dilutions) was added to these wells and incubated for 1 h. Following incubation for 1 h with a secondary HRP-conjugated antibody (1:1000 dilution), specific binding was detected by spectrophotometry using a plate reader (Synergy 4, Bio-tek, Winooski, VT, USA) at 450 nm with a reference wavelength of 655 nm.

### 4.9. Statistical Analysis

Data were expressed as mean ± standard deviation (S.D.). Significance of the differences between controls and experimental groups was determined by one-way ANOVA with Dunnett’s test. *P < 0.05* was considered significant.
